# Conpair: concordance and contamination estimator for matched tumor–normal pairs

**DOI:** 10.1093/bioinformatics/btw389

**Published:** 2016-06-26

**Authors:** Ewa A. Bergmann, Bo-Juen Chen, Kanika Arora, Vladimir Vacic, Michael C. Zody

**Affiliations:** New York Genome Center, New York, NY 10013, USA

## Abstract

**Motivation:** Sequencing of matched tumor and normal samples is the standard study design for reliable detection of somatic alterations. However, even very low levels of cross-sample contamination significantly impact calling of somatic mutations, because contaminant germline variants can be incorrectly interpreted as somatic. There are currently no sequence-only based methods that reliably estimate contamination levels in tumor samples, which frequently display copy number changes. As a solution, we developed Conpair, a tool for detection of sample swaps and cross-individual contamination in whole-genome and whole-exome tumor–normal sequencing experiments.

**Results:** On a ladder of *in silico* contaminated samples, we demonstrated that Conpair reliably measures contamination levels as low as 0.1%, even in presence of copy number changes. We also estimated contamination levels in glioblastoma WGS and WXS tumor–normal datasets from TCGA and showed that they strongly correlate with tumor–normal concordance, as well as with the number of germline variants called as somatic by several widely-used somatic callers.

**Availability and Implementation:** The method is available at: https://github.com/nygenome/conpair.

**Contact:** egrabowska@gmail.com or mczody@nygenome.org

**Supplementary information:** Supplementary data are available at *Bioinformatics* online.

## 1 Introduction

The decreasing cost of high-throughput sequencing allows analysis of larger number of samples than before, which as an unfortunate side effect increases the chances of sample mix-ups and contamination. Cancer studies often jointly analyze matched tumor–normal (T–N) samples in order to detect somatic mutations that are present in the tumor. Even a very low level of cross-individual contamination in the tumor sample may introduce many low allele frequency germline variants that will be interpreted as somatic by somatic variant calling algorithms, resulting in greatly reduced specificity (Supplementary Figure S1). Detecting sample swaps and low level contamination in tumor samples are critical quality control steps that should precede every somatic analysis. However, estimating contamination in tumor samples is confounded by frequent copy number alterations that affect allelic ratio distributions.

VerifyBamID (Jun *et al.*, 2012) and ContEst (Cibulskis *et al.*, 2011) have emerged as standard methods to estimate sample contamination. VerifyBamID maximizes the likelihood of a contamination level in a two-sample mixture model, given the alleles and base qualities, using a grid search over a range of contamination fractions and refining the result using a numerical root-finding method. VerifyBamID provides an accurate measure for contamination in mostly diploid (copy-neutral) samples, however it may interpret copy number-driven allelic imbalance frequently seen in cancer as contamination. ContEst calculates the maximum *a posteriori* estimate of contamination based on the base identities and quality scores from sequencing data, at sites identified on a SNP array to be homozygous. The method can be applied to tumor–normal studies, however ContEst requires additional data from a genotyping array. Alternatively, genotypes of a normal sample called from high coverage (>50×) sequencing data can be used.

We developed Conpair (Concordance/Contamination of paired samples) to robustly detect contamination in cancer studies based on sequence data alone. We show that our method accurately detects contamination levels as low as 0.1% (Supplementary Table S3), even in presence of copy number changes. In contrast to ContEst, our tool also allows verifying concordance between tumor and normal samples and estimating contamination in normal samples. Conpair is ∼50× faster than VerifyBamID and ∼18× faster than ContEst on a 60×/60× WGS pair (Supplementary Figure S11A).

## 2 Methods

Copy number changes, which are frequent in tumor samples, may cause difficulties in estimating contamination levels due to shifting of the expected 50% allelic fraction for heterozygous markers. By using matched normal samples we can robustly detect homozygous markers, which are invariant to copy number changes and are not affected by contamination in the normal sample, and subsequently use them to reliably estimate contamination level in the tumor sample (see Supplementary Methods).

Conpair takes as input a pair of BAM files, the reference genome and a short list of pre-selected highly informative genomic markers that are provided with the tool (see Supplementary Methods), in order to run both concordance verification and contamination estimation. For concordant T–N pairs, Conpair measures contamination first in the normal and then in the tumor sample, using the genotype information from the normal. Conpair employs the statistical model developed by Jun and colleagues (VerifyBamID), but in contrast to VerifyBamID allows for only two alleles and uses a limited set of markers (Supplementary Methods).

## 3 Results

***In silico* contaminated data.** We constructed two independent sets of *in silico* contaminated cancer samples by mixing reads from BAM files from copy number aberrant (Magi *et al.*, 2013) TCGA glioblastoma exomes (Brennan *et al.*, 2013) at a ladder from 0.1% to 95%, yielding a total of 245 samples at 49 different contamination levels (α) in each set. For each sample we estimated α using Conpair, VerifyBamID and ContEst (sequence-only mode). Our results indicate a better agreement between Conpair and the ground truth in both sets (RMSD = 0.0064; 0.009), compared to ContEst (RMSD = 0.0075; 0.0128) or VerifyBamID (RMSD = 0.062; 0.045) (Supplementary Figures S4 and S5).

**TCGA glioblastoma dataset.** After verifying T–N pairing (Supplementary Figure S6), we applied Conpair to 51 WGS and 396 WXS sample pairs from the TCGA glioblastoma study. Since the WGS dataset appeared clean according to Conpair (α: 0.0–0.612%/0–0.905% in the tumor and normal samples respectively), we focused on the less clean WXS dataset (α: 0.008–4.75%/0.014–6.52% in the tumor and normal samples respectively). The WXS dataset consists of 144 T–N pairs that underwent a whole-genome amplification (WGA) library preparation protocol and 252 T–N pairs prepared by exome capture. Conpair, ContEst and VerifyBamID returned similar contamination values for all the normal samples, independently of the library preparation method (Supplementary Figure S7A and C).

For tumor samples, the differences in the values returned by the three programs were substantial. VerifyBamID estimated high α for the majority of the tumor samples. Contamination estimates generated by ContEst were higher, but comparable to Conpair for all samples prepared following exome capture. Conpair and ContEst did not agree on a subset of tumor samples that underwent WGA, for which ContEst detected much higher fractions of contamination (5–10%) (Supplementary Figure S7B and D).

**Fig. 1.**
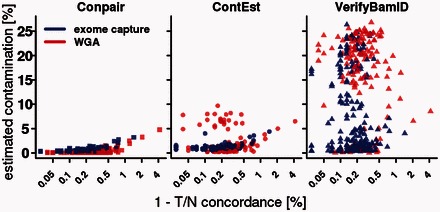
Relationship between tumor–normal discordance values (1 – concordance) and contamination levels detected by Conpair, ContEst and VerifyBamID in a set of TCGA glioblastoma WXS tumor samples. Data shows whole genome amplified samples (red) and exome capture (blue)

To assess which method was more accurate, we correlated the contamination estimates with the T–N concordance values (calculated based only on markers that were homozygous in the normal sample). Tumor samples with T–N concordance values close to 100% cannot be significantly contaminated (Supplementary Figure S2). Based on this fact, we were able to show that VerifyBamID highly overestimated α on the majority of the tumor samples, and ContEst overestimated α on the subset of the WGA samples. The results returned by Conpair show a monotonic dependency between the T–N concordance and contamination values (Fig. 1).

As an independent metric, we also looked at the number of known germline variants called as ‘somatic’ by three somatic callers: MuTect (Cibulskis *et al.*, 2013), LoFreq (Wilm *et al.*, 2012) and Strelka (Saunders *et al.*, 2012). These numbers were strongly correlated with the contamination in the tumor samples returned by Conpair (Spearman *r*: 0.76 [*P*-value = 7.5e–20], 0.75 [5.5e–19], 0.67 [3.7e–14], for variants where α > 0.5%), but not correlated with the estimates returned by ContEst and VerifyBamID (correlations not significant) (Supplementary Figure S8). The obtained results suggest that Conpair is more robust in estimating contamination levels in the light of different library preparation methods.

## Supplementary Material

Supplementary Data
