# Supplementary material for: Conpair: concordance and contamination estimator for matched tumor–normal pairs
Source: Bioinformatics. 2016 Jun 26;32(20):3196–8. doi: 10.1093/bioinformatics/btw389 (PMC5048070; doi:10.1093/bioinformatics/btw389)
Supplement: Supplementary Data [file btw389_supplementary_data.zip › Conpair_Supplementary_Methods.pdf]

Supplementary Methods for Conpair: concordance and contamination estimator for matched tumor – normal pairs

Ewa A. Bergmann, Bo-Juen Chen, Kanika Arora, Vladimir Vacic and Michael C. Zody

Contamination level and number of somatic variants

We called somatic SNVs using MuTect (Cibulskis et al., 2013) version 1.1.4, Strelka (Saunders et al., 2012) version 1.1.13, and Lofreq (Wilm et al., 2012) version 2.0.0, with default parameters.

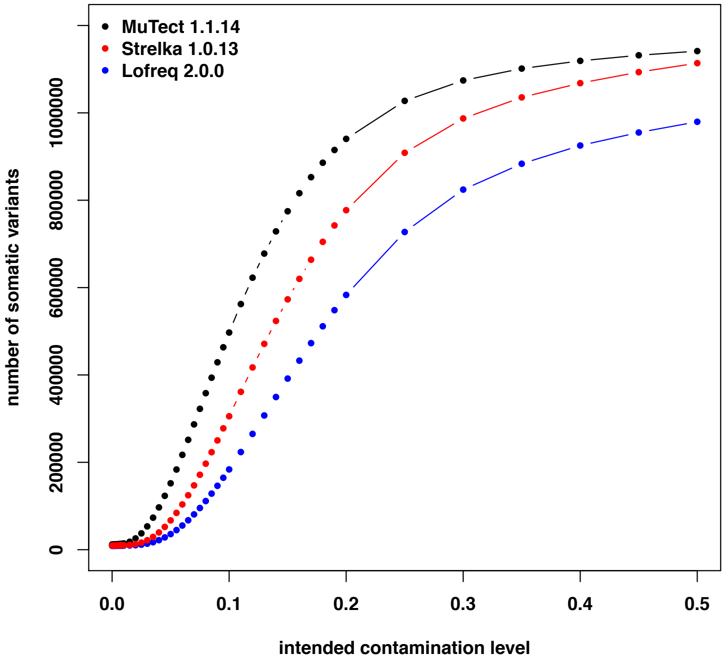

Supplementary Figure 1: The number of somatic variants called by three independent methods (MuTect, Strelka, Lofreq) as a function of contamination level in a WXS tumor sample with a matched normal.

Selection of informative genomic markers (GRCh37/hg19)

The selected 7387 markers meet the following criteria:

- SNVs (easier to genotype from sequencing data)
- exonic (to allow comparison of exome and WGS samples)
- located on autosomes (to have estimates that are consistent across both sexes)
- minor allele frequency (MAF) ≥ 40%, estimated across all populations in the 1000 Genomes Project (Consortium, 2012), phase 3 dataset*
- linkage disequilibrium (LD) between any two markers < 0.8

*) We used the entire 1000 Genomes, phase 3 dataset. It consists of 2535 individuals from 26 different populations. More details can be found on: http://www.1000genomes.org/announcements/initial-phase-3-variant-list-and-phased-genotypes-2014-06-24/

Calculating genotypes

To calculate genotypes we use the method described previously by Heng Li (Heng Li, 2010):

Given k reads and assuming that only two alleles are possible for a given site:

$$D = (b_1, b_2, \dots, b_j) = (A, \dots, A, B, \dots, B)$$

$$l : \; number\ of\ occurances\ of\ A$$

$$k - l : number\ of\ occurances\ of B$$

Given that the j-th base is associated with error rare e_j:

$$P(D|AA) = \prod_{j=1}^{l} \epsilon_j \prod_{j=l+1}^{k} (1 - \epsilon_j)$$

$$P(D|BB) = \prod_{j=l+1}^{k} (1 - \epsilon_j) \prod_{j=j=l+1}^{k} \epsilon_j$$

$$P(D|AB) = 2^{-k}$$

We normalize the results and apply priors calculated based on marker's MAF.

Concordance value between two samples

The expected concordance between samples coming from the same individual is 100% and can be slightly lower in case of sequencing errors, mosaicism, contamination and/or copy number events (or other events leading to imbalance of 50/50 ratio of heterozygous alleles). When samples come from unrelated individuals the expected concordance rate (based on applying the Hardy-Weinberg equation and integrating across MAFs of all preselected markers) is in theory close to 37.8% assuming independence of markers, good coverage, no copy number changes and no sequencing errors. In practice, we observe a slightly higher median discordance value (close to 40%).

Conpair provides an option to use only homozygous markers ('--normal_homozygous_markers_only') for tumor – normal concordance verification. Homozygous markers are selected based on the normal sample. This approach eliminates the effects of copy number variation in the tumor sample and potential contamination in the normal sample on level of concordance between correctly matched samples. However, when this option is used, the concordance rate is much more sensitive to contamination (decreases quickly when contamination increases) as shown in Supplementary Figure 2. Also, the expected concordance between two unrelated samples is ~27%. We again observe slightly higher values due to the fact that the markers are not fully independent.

In general, concordance level between two samples depends on multiple factors, such as choice of genomic markers, depth of coverage, sequencing error rate, presence of contamination, copy number changes, etc. Based on our results (Supplementary Table 2, Supplementary Figure 6), we recommend using only homozygous markers ('--

normal_homozygous_markers_only') and the following cut-offs for Illumina sequencing (assuming at least 20% of all markers are covered >10x in both samples):
>80% means that two samples come from the same individual,
<50% means that two samples are not related or highly contaminated,
50-80% requires an additional investigation.

Intermediate values could be a sign that two samples are not concordant, but they are coming from related individuals (siblings, parent-child, etc.).

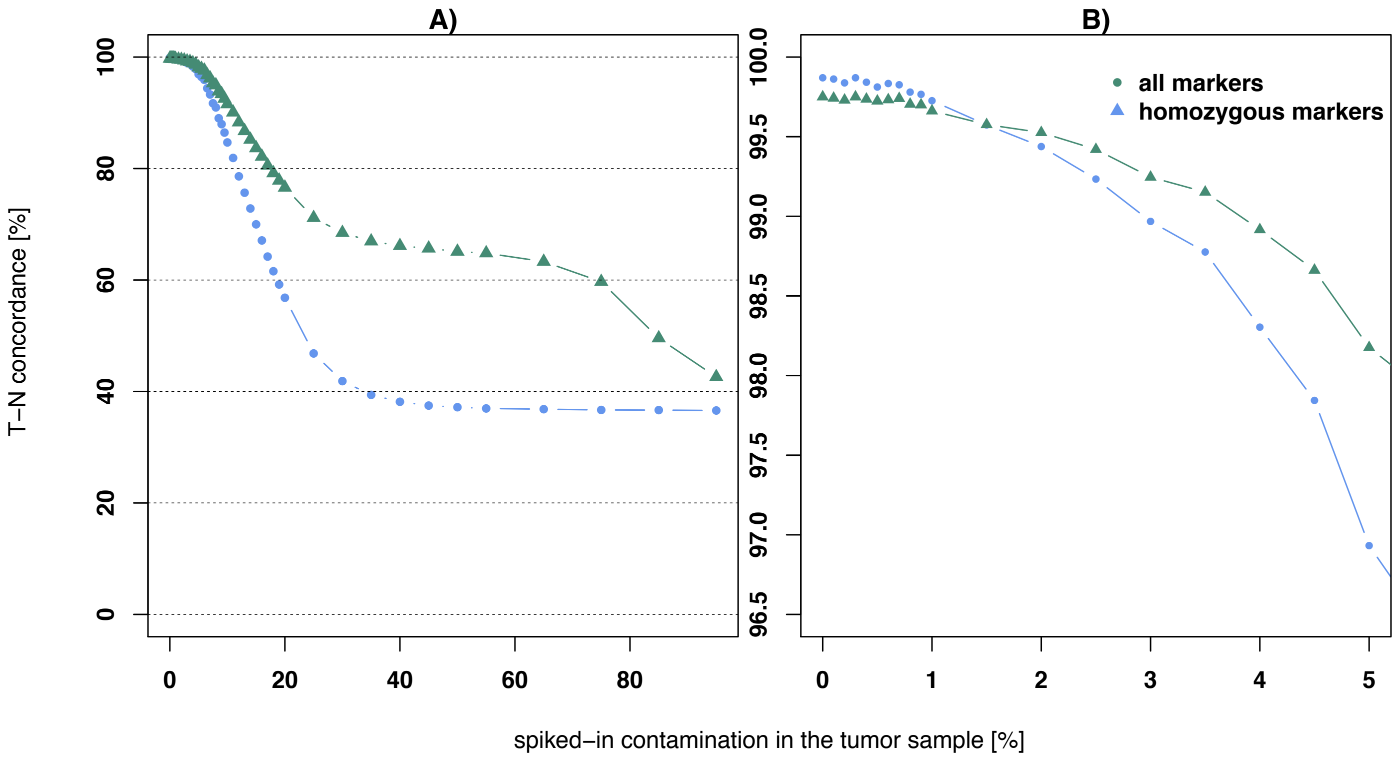

Supplementary Figure 2: Relationship between contamination level in a tumor sample and concordance level. As contamination level increases, concordance decreases more rapidly when normal homozygous markers (blue points) are being used, compared to the use of homozygous and heterozygous markers (green triangles). Shown values are means of 5 independent experiments. Standard errors are too small to be visible on the plot. Panel A) shows spike-in contamination range: 0-95% and B) zoom-in: 0-5%.

Contamination model

Conpair utilizes the probabilistic method developed by Jun and colleagues (Jun *et al*., 2012) (VerifyBamID), in which a two-sample mixture model is used to calculate the conditional likelihood of observing allele *b* at site *i* for read *j* given the original (g_i^1) and contaminating (g_i^2) genotypes, a binary base quality error indicator (e_{ij}) and contamination level (α):

$$\mathrm{P}\left(b_{ij}|g_i^1, g_i^2, e_{ij}; \alpha\right) = (1-\alpha)P\left(b_{ij}\middle|g_i^1, e_{ij}\right) + \alpha P\left(b_{ij}\middle|g_i^2, e_{ij}\right)$$

When the prior genotype data is not available, g_i^1 and g_i^2 are unknown, and $P(g_i^1)$ and $P(g_i^2)$ is taken from the population frequency data assuming Hardy-Weinberg equilibrium.
In case of the tumor samples, the $P(g_i^1)$ is calculated based on the data retrieved from the normal sample.

$$\mathcal{L}(\alpha) = \prod_{i=1}^{M}\sum_{g_i^1}\sum_{g_i^2}\left\{\prod_{j=1}^{R_i}\sum_{e_{ij}}\left((1-\alpha)P\left(b_{ij}\middle|g_i^1, e_{ij}\right) + \alpha P\left(b_{ij}\middle|g_i^2, e_{ij}\right)\right)P(e_{ij})\right\}P(g_i^2)P(g_i^1)$$

Next, similarly to VerifyBamID, we maximize the likelihood function, first using a grid search and then applying Brent's algorithm. α that maximizes the likelihood function is the estimated level of contamination.

Unlike VerifyBamID, Conpair uses a small preselected set of informative genomic markers, which greatly decreases the run time. Our choice of markers allows us to assume that only two alleles (A, B) are possible for each marker. Under the infinite sites model, presence of a third allele will be very rare and is more likely to be a sequencing error. By focusing only on the counts of the two common alleles, we are effectively reducing the influence of sequencing substitution errors by ⅔.

**True genotype g_i**	**Base calling error event e_{ij}**	**Pr(b_{ij}=A)**	**Pr(b_{ij}=B)**	**Pr(b_{ij}=E)**
g_i=AA	e_{ij}=0	1	0	0
	e_{ij}=1	0	1	0
g_i=AB	e_{ij}=0	0.5	0.5	0
	e_{ij}=1	0.5	0.5	0
g_i=BB	e_{ij}=0	0	1	0
	e_{ij}=1	1	0	0

Supplementary Table 1: Conditional probability P($b_{ij}|e_{ij}g_i$) of read b_{ij} given true genotype g_i, and Read Error e_{ij}. Our model, in contrast to verifyBamID allows only for two alleles (A, B). Alleles E, different than A or B, are considered to be sequencing errors.

The idea behind Conpair is based on an observation that homozygous markers are not sensitive to copy number changes because neither amplifications nor deletions can perturb the 100% allelic fractions of such markers. Cross-individual contamination however, may lower the 100% allelic fraction. Conpair, by using matched normal samples (that in contrast to tumor samples are rarely affected by copy number changes), robustly identifies homozygous markers and uses them to accurately estimate contamination in the tumor sample.

The entire Conpair procedure is as follows:

1) Compute concordance for all pairs of samples in a project using the set of markers. Verify concordance between a tumor – normal pair.
2) Compute contamination estimate for a normal sample.
3) Find homozygous markers in the normal.
4) Compute contamination in the tumor sample using homozygous markers only.

In silico contaminated data

To show that our method accurately estimates contamination levels, we constructed both cancer and non-cancer *in silico* contaminated samples by randomly combining known fractions (from 0.1% to 95%) of aligned sequence reads of a "contaminated" sample with complementing fractions of a "contaminating" sample.

In silico contaminated data: non-cancer

Estimating contamination in non-cancer samples is not the main goal of Conpair, since there are other great tools (VerifyBamID) that successfully address this issue., We nevertheless wanted to demonstrate that Conpair performs reasonably well on non-cancer (copy-neutral) samples.

As non-cancer samples we used two unrelated HapMap CEU samples (The 1000 Genomes Project Consortium, 2010): NA12891 (contaminated) and NA12892 (contaminating). We generated a set of 245 contaminated NA12891 samples at 49 different contamination levels.

For each sample we estimated cross-individual contamination level using our method, VerifyBamID and ContEst, last two tools were run in sequence-only mode. For Conpair and ContEst we used the original (uncontaminated) NA12891 as "normal" and each of the *in silico* contaminated samples as "tumors" to satisfy the matched samples requirement.

Since VerifyBamID measures contamination in respect to the population background, the reported contaminating fractions can never exceed 50%. For contamination larger than 50%, VerifyBamID reports 1 – contamination level. For this reason we limited computation of the root-mean-square deviation (RMSD) of the three methods to the 0%-50% range. For practical purposes all three methods performed pretty much identically, very accurately estimating spiked-in contamination levels (RSMD: 0.0063, 0.0087, 0.0122 for Conpair, VerifyBamID and ContEst respectively), as shown in Supplementary Figure 3.

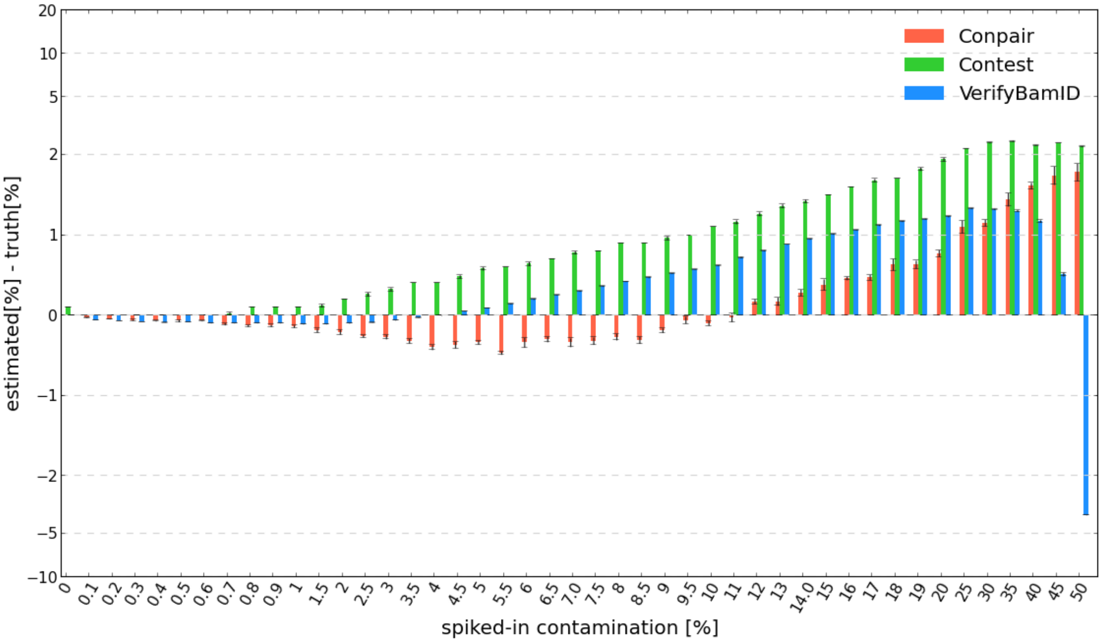

Supplementary Figure 3: *In silico* 60x whole genome sample NA12891 contaminated with NA12892: spiked-in contamination levels range from 0.1% to 95% (for a better resolution we show here 0-50%). The contamination level was estimated using Conpair (red), ContEst (green) and verifyBamID (blue). We plotted the mean values from 5 independent *in silico* contamination experiments of the difference between the estimated and targeted contamination levels. The standard error of the mean is marked with black bars. Y-axis is shown in the logarithmic scale. The uniformity of the results returned by ContEst in low fractions of α are due to the precision level ContEst uses (0.1%).

In silico contaminated data: cancer

To demonstrate that Conpair works robustly on samples with copy number changes, similarly to the HapMap contamination, we generated two sets of 245 *in silico* contaminated cancer samples at 49 different contamination levels (from 0.1% to 95%). In the first set TCGA-06-0168-01A-01D-1491-08 – a rearranged tumor sample (Supplementary Figure 4A) from the TCGA glioblastoma WXS study was *in silico* contaminated with another tumor sample from the same study: TCGA-06-0132-01A-02D-1491-08 (Supplementary Fig. 4B). In the second set another tumor sample: TCGA-06-0154-01A-03D-1491-08 (Supplementary Fig. 5A) was contaminated with TCGA-06-0185-01A-01D-1491-08 (Supplementary Fig. 5B). For each sample we estimated cross-individual contamination level using Conpair, ContEst and VerifyBamID. Both Conpair and ContEst were able to accurately predict levels of contamination in the presence of copy number changes in the tumor samples. ContEst overestimated contamination level when contamination is <50%, while Conpair slightly underestimated it. VerifyBamID was the least accurate because of its sensitivity to copy number changes (Supplementary Figure 4C, 5C).

A)

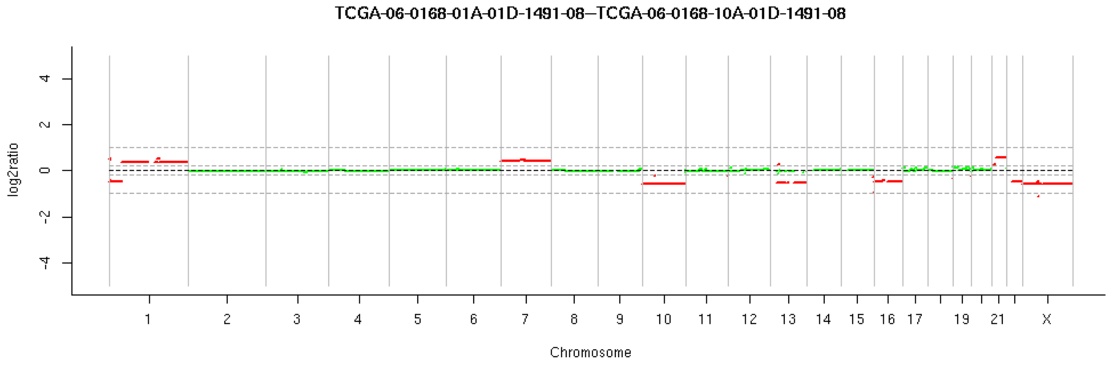

B)

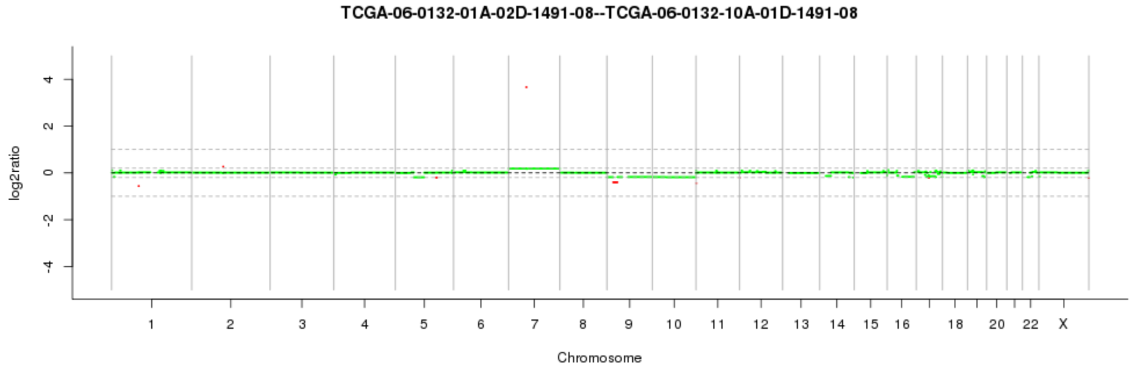

C)

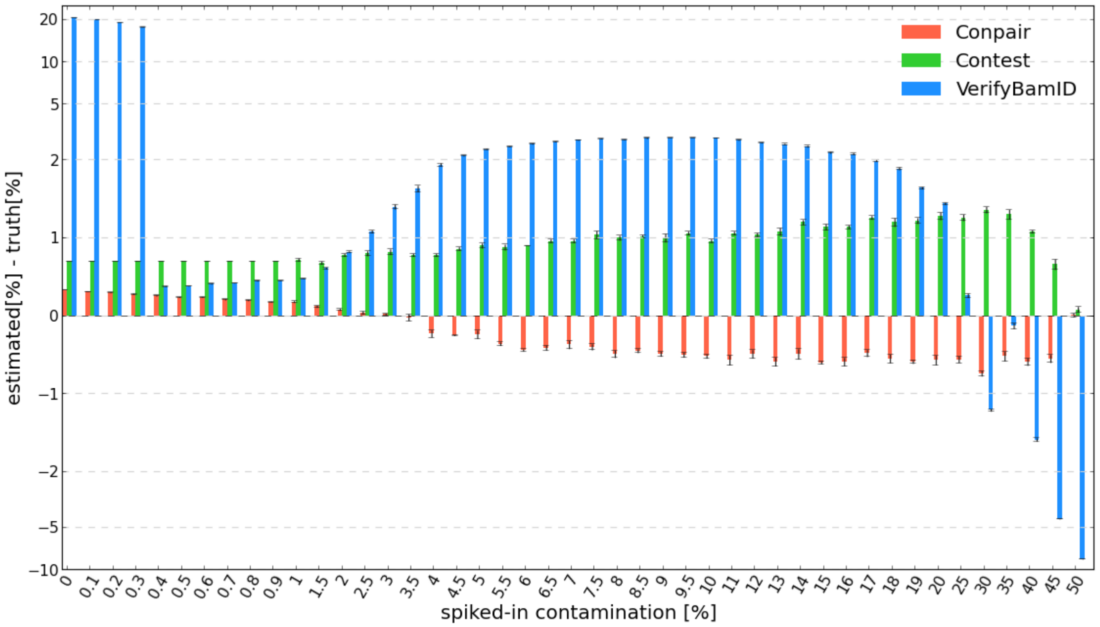

Supplementary Figure 4: A) Original CNV profile of TCGA-06-0168-01A-01D-1491-08 [Excavator], WXS sample, showing whole arm and focal deletions and amplifications; B) Original CNV profile of the contaminating sample (TCGA-06-0132-01A-02D-1491-08) [Excavator]; C) *in silico* contaminated TCGA-06-0168-01A-01D-1491-08 (WXS) with TCGA-06-0132-01A-02D-1491-08: spiked-in contamination levels range from 0.1% to 95% (for a better resolution we show here 0-50%). The contamination level was estimated using Conpair (red) and ContEst (green) and VerifyBamID (blue). We plotted the mean values from 5 independent *in silico* contamination experiments of the difference between the estimated and targeted contamination levels. The standard error of the mean is marked with black bars.

A) 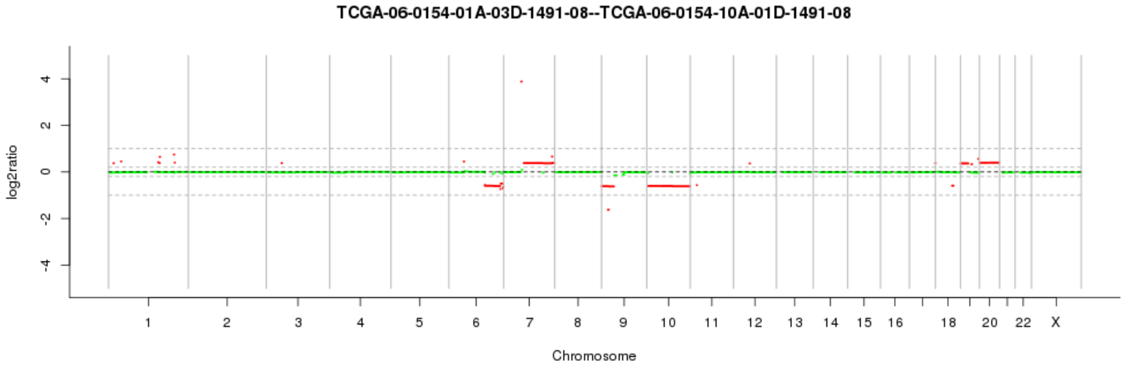

B) 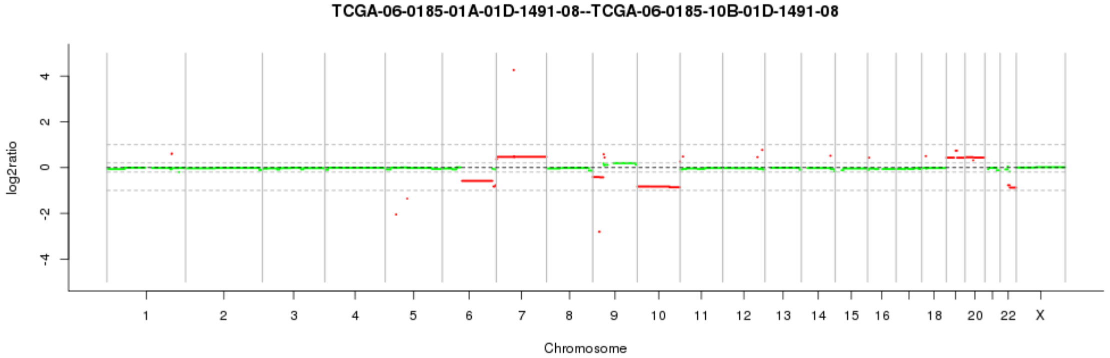

C) 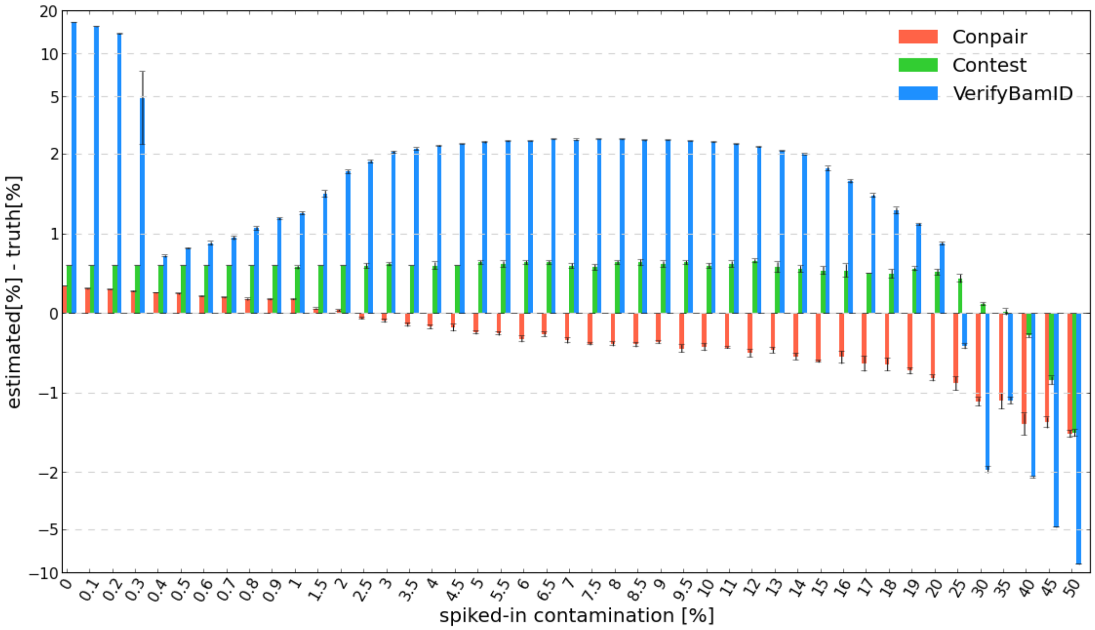

Supplementary Figure. 5: A) Original CNV profile of TCGA-06-0154-01A-03D-1491-08 [Excavator], WXS sample, showing whole arm and focal deletions and amplifications; B) Original CNV profile of the contaminating sample (TCGA-06-0185-01A-01D-1491-08) [Excavator]; C) *in silico* contaminated TCGA-06-0154-01A-03D-1491-08 (WXS) with TCGA-06-0185-01A-01D-1491-08: spiked-in contamination levels range from 0.1% to 95% (for a better resolution we show here 0-50%). The contamination level was estimated using Conpair (red) and ContEst (green) and VerifyBamID (blue). We plotted the mean values from 5 independent *in silico* contamination experiments of the difference between the estimated and targeted contamination levels. The standard error of the mean is marked with black bars.

TCGA glioblastoma dataset

The TCGA glioblastoma dataset (Brennan et al., 2013) consists of 51 WGS and 396 WXS tumor-normal (T/N) pairs. Whole Genome Amplification (WGA) was used in library preparation for 144 WXS pairs, the remaining 252 WXS pairs underwent regular DNA library preparation procedure.

Based on a detailed analysis we excluded 3 T-N pairs from the further investigation:

1. T: TCGA-06-0178-01A-01D, N: TCGA-06-0178-10B-01D-1491-08: the samples have been swapped.
2. T: TCGA-08-0386-11A-01D, N: TCGA-08-0386-11A-01D-1492-08: the normal sample is highly infiltrated with the tumor DNA.
3. T: TCGA-12-0772-01A-01W, N: TCGA-12-0772-10A-01W: both samples highly contaminated.

Concordance

We assessed accuracy of concordance verification by applying Conpair to all possible tumor – normal pairs from the TCGA glioblastoma WGS and WES datasets. The concordance between matching samples was in the 99.52-99.95% range for the WGS samples, and in 91.58-100% for the WXS samples, using the homozygous markers only ('--normal_homozygous_markers_only'). Discordant samples had 25.47-42.84% and 21.17-46.58% marker concordance levels for WGS and WXS datasets respectively (Supplementary Tab. 2, Supplementary Fig. 6).

**study**	**# comparisons**	**min**	**max**	**mean**	**median**	**1st qu**	**3rd qu**
**TCGA GBM WGS-matched**	51	99.52	99.95	99.82	99.82	99.78	99.87
**TCGA GBM WGS-unmatched**	1860	25.47	42.84	34.59	34.92	33.87	35.82
**TCGA GBM WXS-matched**	559	91.58	100.0	99.72	99.79	99.71	99.86
**TCGA GBM WXS-unmatched**	159029	21.17	46.58	34.23	35.02	33.24	36.35

Supplementary Table 2: The summary of Conpair concordance results for the TCGA GBM WGS and WXS studies.

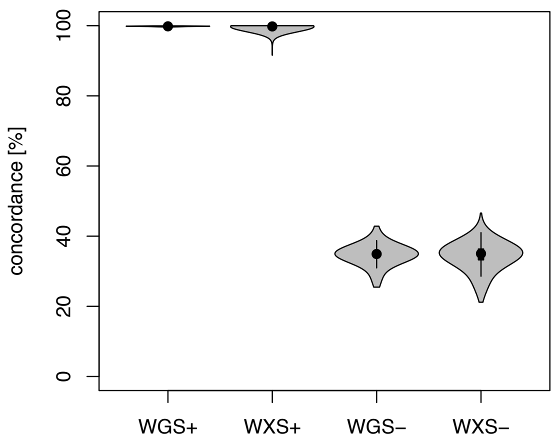

Supplementary Fig. 6: Violin plots of the Conpair concordance values for TCGA GBM WGS & WXS. Concordance values robustly separate matched from unmatched sample pairs.

Contamination

Accurate estimation of contamination level is less challenging in normal (copy-neutral) samples than in highly rearranged tumor samples. Contamination levels estimated by Conpair, ContEst and VerifyBamID were highly concordant for the normal samples (Supplementary Fig. 7A, C) and substantially discordant for the tumor samples (Supplementary Fig. 7B, D).

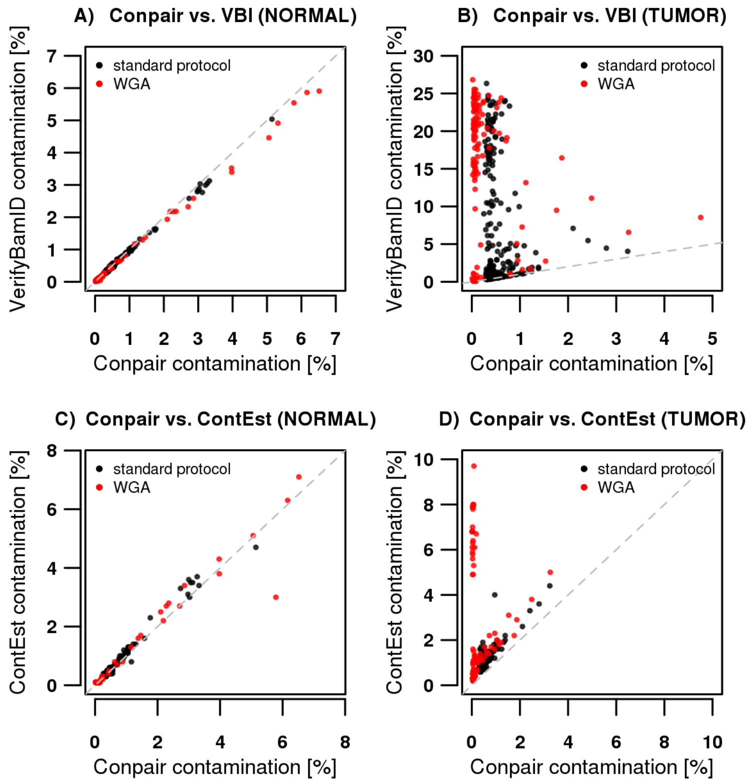

Supplementary Figure 7: Relationship between contamination levels detected in TCGA glioblastoma WXS tumor and normal samples by A) Conpair and VerifyBamID (normal samples), B) Conpair and VerifyBamID (tmor samples) C) Conpair and ContEst (normal samples) D) Conpair and ContEst (tumor samples). Data shows whole genome amplified samples (red) and exome capture (black).

As we have shown before (Supplementary Figure 1) the number of germline variants called as somatic increases with tumor sample contamination. The number of such mutations mostly depends on the contamination level and does not vary much from sample to sample (the number of variants with different genotypes between two unrelated samples is normally distributed). We use the number of known germline mutations (AF>1% in 1000 Genomes and ExAC, AF >5% in 1000 Genomes and ExAC for DNMT3A, TET2, JAK2, ASXL1, TP53, GNAS, PPM1D, BCORL1, SF3B1) called as somatic (by MuTect, LoFreq and Strelka) as a reliable measure of contamination fraction in a tumor sample (Supplementary Fig. 8).

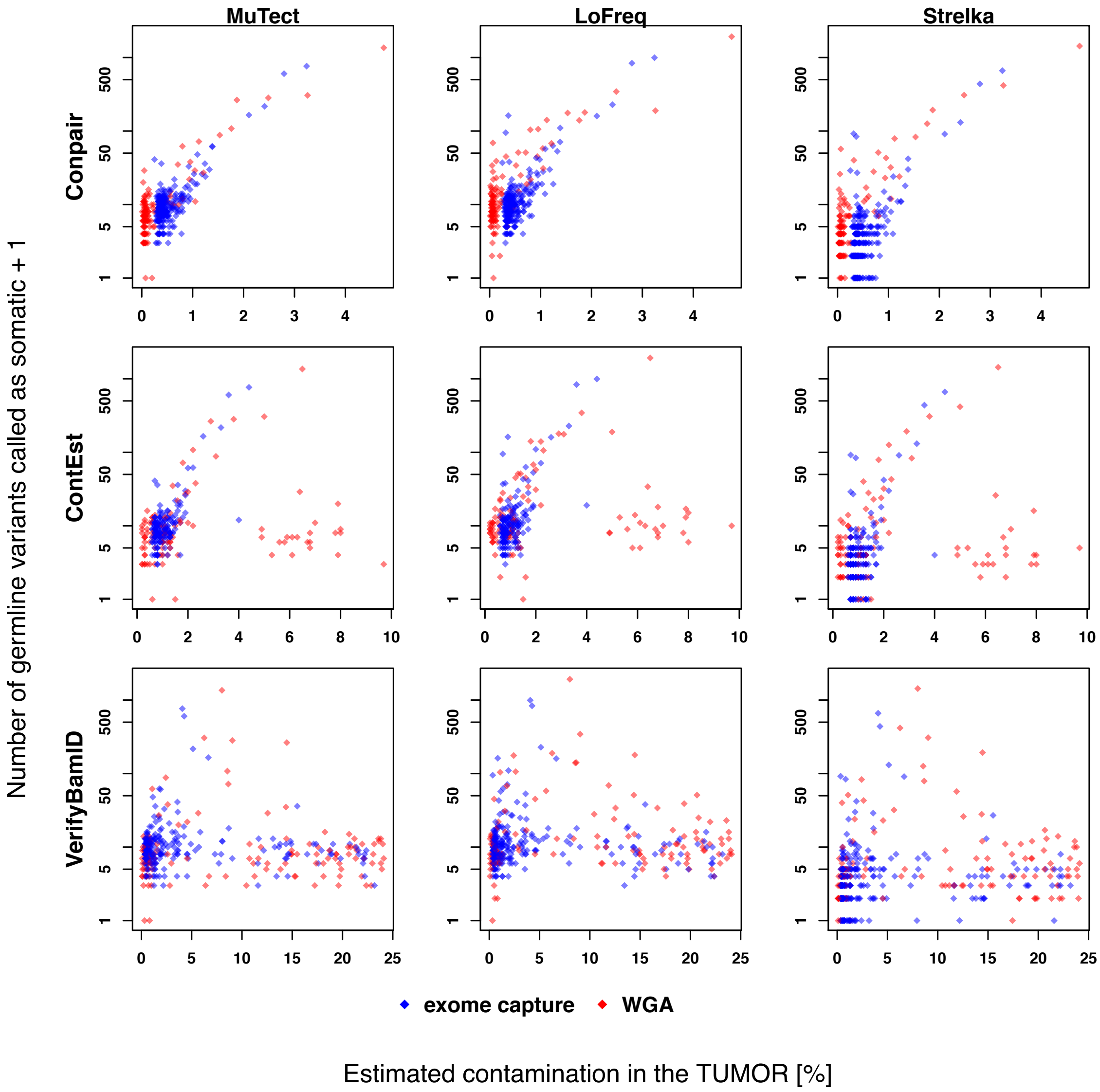

Supplementary Figure 8: TCGA glioblastoma WXS dataset: Relation between the estimated contamination levels by Conpair (top panel), ContEst (middle panel) and VerifyBamID (bottom panel) and the number of known germline called as somatic by three independent methods: MuTect, LoFreq, Strelka. The samples that underwent the WGA library preparation protocol are shown in red and the samples prepared following the standard exome capture protocol are shown in blue.

We further show that contamination estimations returned by Conpair are strongly correlated with the number of germline variants called as somatic based on Spearman correlation. To reduce the noise in the low range of α, we tested different α cut-offs (discarding samples with contamination level below the cut-off) to calculate Spearman correlation coefficients (Supplementary Fig. 9). Conpair estimates correlate better than those generated by ContEst and VerifyBamID, regardless of the somatic calling method used or applied cut-off.

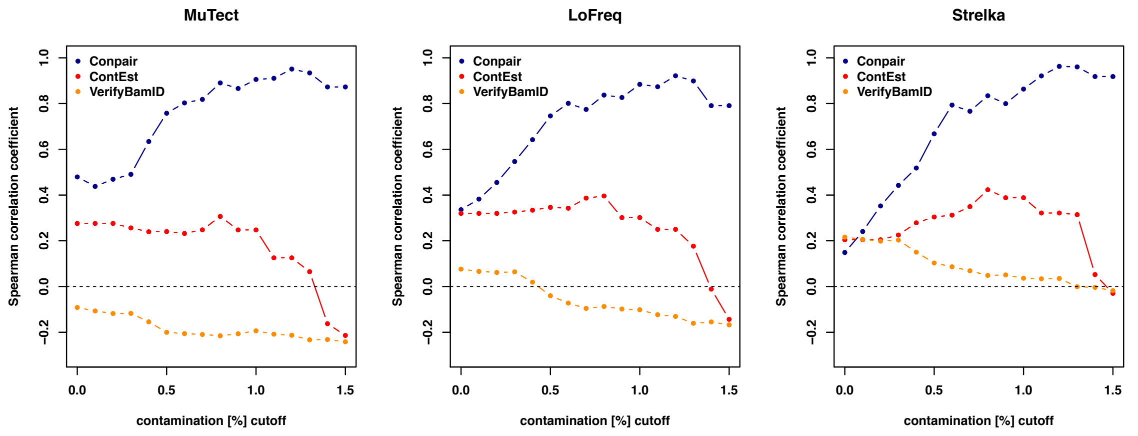

Supplementary Figure 9: Spearman correlation coefficients between estimated contamination level (Conpair: blue, ContEst: red, VerifyBamID: orange) and the number of germline variants called as somatic by MuTect, LoFreq and Strelka. The plots show how the correlation changes when different contamination cut-offs (discarding samples with contamination levels between the specified cut-off) are applied.

Accuracy of Conpair

The challenge of estimating the accuracy of any contamination method lies in the technical problem of assessing the initial (real) contamination level of a tumor sample. For this reason we used two approaches that gave as a concordance accuracy level:

1. Using the “cleanest” (based on Conpair estimates) TCGA glioblastoma WGS sample, and performing *in silico* contaminations in 3 independent repeats.
2. Using the gold standard NA12891 sample, which we strongly believe is uncontaminated (0.0% estimated contamination returned by VerifyBamID, ContEst and Conpair) and *in silico* contaminating it with known fraction of NA12892.

For all experiment replicates Conpair was able to clearly distinguish between 0% ans 0.1% contamination. Based on the results (summarized in Supplementary Tab. 3) we estimate the accuracy of Conpair being on the 0.01% level.

targeted α [%]	TCGA glioblastoma WGS	NA12891
0.0	0.006 ± 0.0	0.0 ± 0.0
0.1	0.133 ± 0.0062	0.071 ± 0.0035
0.2	0.231 ± 0.0093	0.145 ± 0.0007
0.3	0.358 ± 0.0105	0.244 ± 0.0084
0.4	0.428 ± 0.004	0.332 ± 0.0054
0.5	0.542 ± 0.0101	0.433 ± 0.0154
0.6	0.688 ± 0.0102	0.533 ± 0.0043
0.7	0.81 ± 0.0115	0.585 ± 0.0129

0.8	0.91 ± 0.0067	0.67 ± 0.0134
0.9	1.002 ± 0.0187	0.774 ± 0.015
1.0	1.063 ± 0.0233	0.856 ± 0.0154
1.5	1.655 ± 0.0317	1.305 ± 0.0263
2.0	2.244 ± 0.035	1.785 ± 0.0286
2.5	2.794 ± 0.0744	2.242 ± 0.0164
3	3.325 ± 0.024	2.728 ± 0.0274
3.5	4.005 ± 0.0187	3.184 ± 0.0292
4	4.443 ± 0.0192	3.609 ± 0.0309
4.5	5.064 ± 0.0178	4.132 ± 0.0404
5	5.647 ± 0.0332	4.657 ± 0.0239

Supplementary Table 3: Accuracy of Conpair based on two different data sets: 1) TCGA glioblastoma WGS sample (TCGA-19-2624-01A-01D-1495-08 contaminated with TCGA-19-2620-01A-01D-1495-08 2) NA12891 (contaminated with NA12892). We show mean values and standard errors of the mean coming from three independent *in silico* contamination experiments.

VerifyBamID algorithm instability in copy number aberrant samples

Since VerifyBamID uses a deviation from 100/0 (homozygous positions) and 50/50 (heterozygous positions) allelic ratios to estimate α, we investigated whether there is a relationship between the extent of copy number changes and contamination levels returned by the method.

We used Excavator (Magi et al., 2013) to detect amplified/deleted regions. Regions with log2 value above 0.2 were classified as amplified, below -0.2 value as deleted. We observed that for samples with relatively low fraction of the genome being amplified/deleted, VerifyBamID returned low contamination values. When more than ~7% of the genome was affected by copy number changes, the VerifyBamID became unstable and often highly overestimated α (Supplementary Fig. 10).

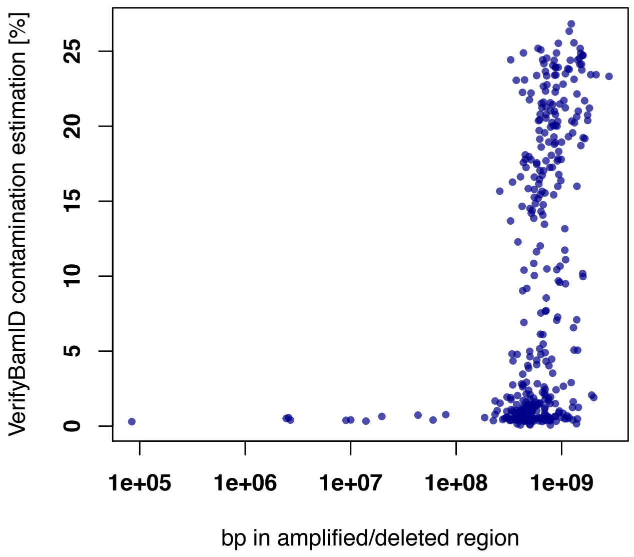

Supplementary Figure 10: Relationship between the extent of copy number changes and contamination estimates returned by VerifyBamID for the TCGA glioblastoma WXS dataset (both WGA and standard protocols).

Run time

The pre-selection of the genomic markers allows for a greatly reduced running time of Conpair.

We measured running time for TCGA glioblastoma WGS (30x) and WXS (120x), and for non-cancer NA12891 (60x) for all three methods. The input files and all the steps included in the run time calculation are presented in Supplementary Tab. 4.

METHOD	INPUT FILE(S)	STEPS INCLUDED IN RUNTIME CALCULATION
Conpair	Tumor_BAM, Normal_BAM	1. Pileup generation<br>2. Concordance verification<br>3. Contamination estimation for both TUMOR and NORMAL
ContEst	Tumor_BAM, Normal_VCF	1. Contamination estimation for TUMOR
VerifyBamID	Tumor_BAM, population_VCF	1. Contamination estimation for TUMOR

Supplementary Table.4: Input files and steps included in calculation of the run times for three methods: Conpair, ContEst and VerifyBamID.

Since ContEst requires that the coverage in normal samples is > 50x, we could not measured ContEst performance on the TCGA glioblastoma WGS dataset.

In contrast to both VerifyBamID and ContEst, Conpair also returns the estimated contamination level in the normal sample.

In terms of running time, Conpair outperforms two other methods. It runs >18x faster than ContEst and ~50x faster than VerifyBamID one the WGS 60x and WGS 30x dataset (Supplementary Fig. 11A, B). When measured on WXS samples, Conpair was non-significantly slower than ContEst and VerifyBamID, mostly due to the preceding pileup generation step (Supplementary Fig. 11C).

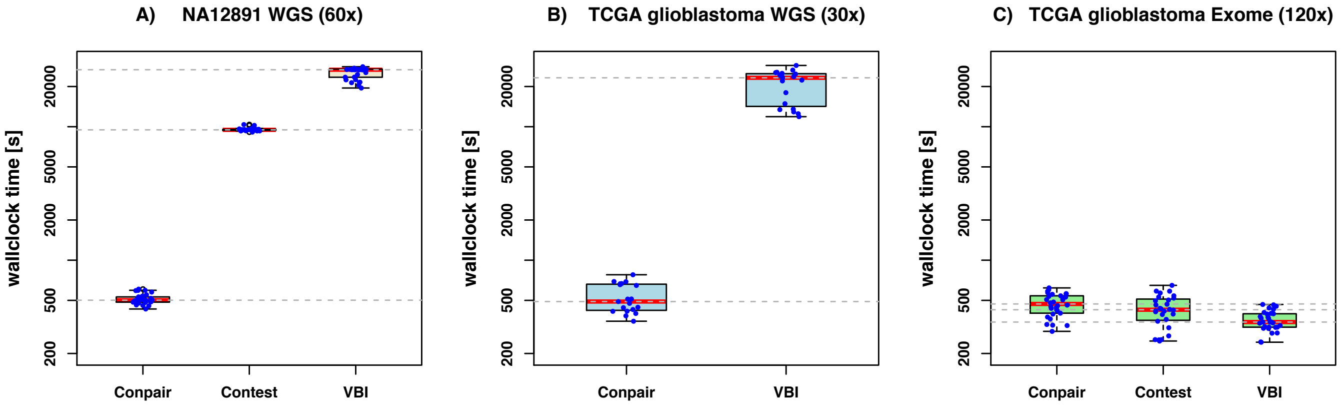

Supplementary Figure 11: Running time [s] comparison for 3 methods: Conpair, ContEst, VerifyBamID. A) WGS (60x) *in silico* contaminated NA12891 sample B) TCGA glioblastoma WGS (30x) C) TCGA glioblastoma Exome (120x). Jobs were running on a single core. The results are shown in the logarithmic scale.

Applying the set of markers provided with Conpair to ContEst/VerifyBamID

The selection of markers allows Conpair to focus on the most informative positions in the human genome and reduce the impact of sequencing substitution errors by 2/3. We wanted to verify if the same set of markers would improve performance of ContEst and VerifyBamID.

We used three *in silico* contaminated data sets previously described (one non-cancer NA12891 and two TCGA glioblastoma WXS). For the non-cancer data, applying Conpair's genomic markers does not seem to change the contamination estimations (Supp. Fig. 12A). However, when we looked at the tumor WXS data, the results returned by VerifyBamID were less accurate and ContEst seems to improve its accuracy in case of the first date set, but the trend was not so clear in the second data set (Supp. Fig. 12B, C).

A)

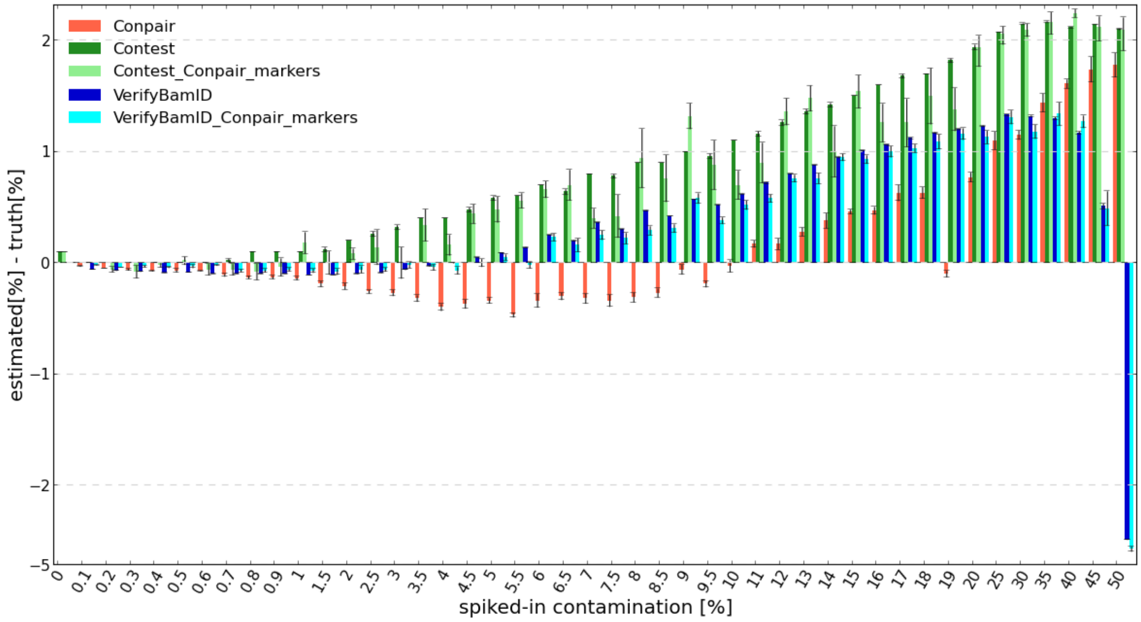

B)

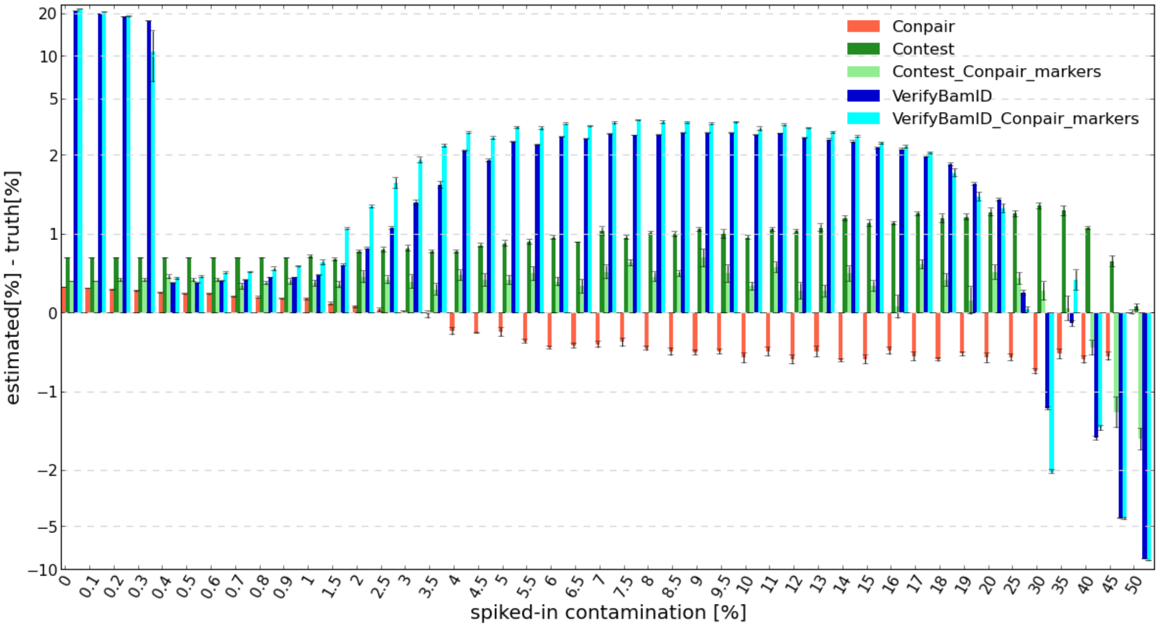

C)

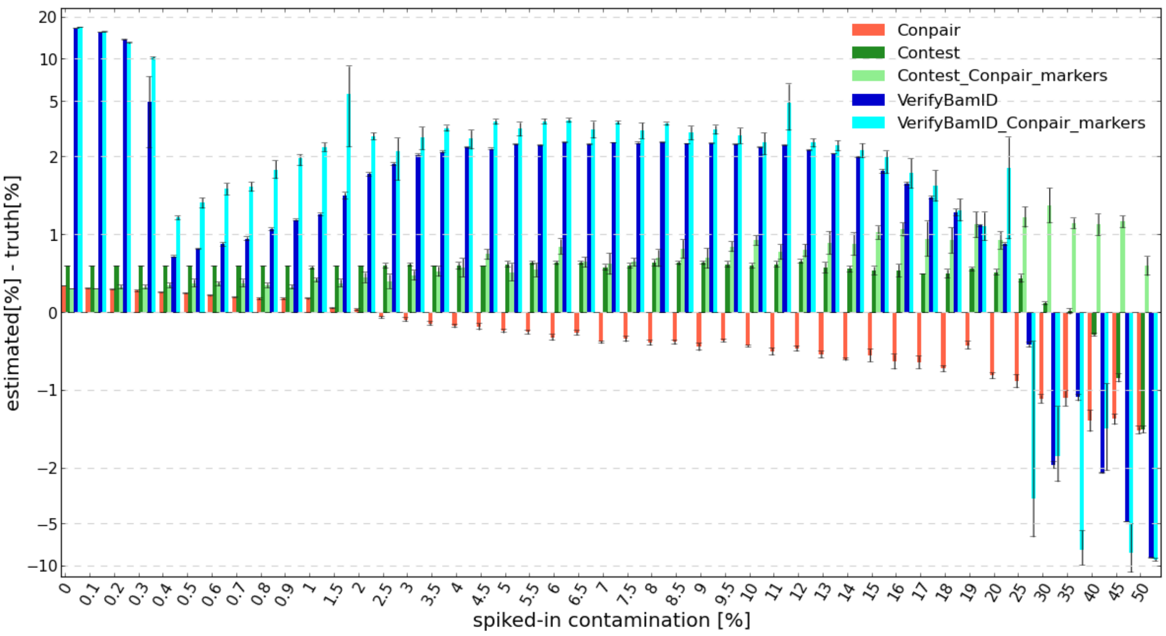

Supplementary Figure 12: Change of accuarcy of ContEst andVerifyBamID when the marker's provided by Conpair were used. We plotted the mean values from 5 independent *in silico* contamination experiments. The standard error of the mean is marked with black bars. A) In silico contaminated WGS 60x NA12891 sample with NA12892 B) *in silico* contaminated TCGA-06-0168-01A-01D-1491-08 (WXS) with TCGA-06-0132-01A-02D-1491-08 C) *in silico* contaminated TCGA-06-0154-01A-03D-1491-08 (WXS) with TCGA-06-0185-01A-01D-1491-08.

Limitations of Conpair

Conpair in the current form is dedicated for tumor – matched normal WGS and WXS human cancer studies, but it can be easily extended to non-human organisms.

Conpair relies on germline markers to estimate contamination levels. Therefore, in cases where the targeted sample is contaminated with DNA coming from a relative, the contamination level will be underestimated. However, given that our method is intended for cancer studies, the probability of having two related samples in any given cohort is relatively small.

Conpair has not been tested on low coverage data. We assume that the mean coverage in both the normal and the tumor is at least 20x.

References:

Brennan, C.W. *et al*. (2013). The Somatic Genomic Landscape of Glioblastoma. Cell *155*, 462–477.

Cibulskis, K. *et al*. (2011). ContEst: estimating cross-contamination of human samples in next-generation sequencing data. Bioinforma. Oxf. Engl. *27*, 2601–2602.

Cibulskis, K. *et al*. (2013). Sensitive detection of somatic point mutations in impure and heterogeneous cancer samples. Nat. Biotechnol. *31*, 213–219.

Consortium, T. 1000 G.P. (2012). An integrated map of genetic variation from 1,092 human genomes. Nature *491*, 56–65.

Heng, L. (2010) Mathematical Notes on SAMtools Algorithms, www.broadinstitute.org/gatk/media/docs/Samtools.pdf

Jun, G *et al*. (2012). Detecting and Estimating Contamination of Human DNA Samples in Sequencing and Array-Based Genotype Data. Am. J. Hum. Genet. *91*, 839–848.

Magi, A. *et al*. (2013). EXCAVATOR: detecting copy number variants from whole-exome sequencing data. Genome Biol. *14*, R120.

McKenna, A. *et al*. (2010). The Genome Analysis Toolkit: a MapReduce framework for analyzing next-generation DNA sequencing data. Genome Res. *20*, 1297–1303.

Saunders, C.T. *et al*. (2012). Strelka: accurate somatic small-variant calling from sequenced tumor-normal sample pairs. Bioinforma. Oxf. Engl. *28*, 1811–1817.

The 1000 Genomes Project Consortium (2010). A map of human genome variation from population scale sequencing. Nature *467*, 1061–1073.

Wilm, A. *et al*. (2012). LoFreq: a sequence-quality aware, ultra-sensitive variant caller for uncovering cell-population heterogeneity from high-throughput sequencing datasets. Nucleic Acids Res. *40*, 11189–11201.
